# Inhibition of the MAPK/c-Jun-EGR1 Pathway Decreases Photoreceptor Cell Death in the *rd1* Mouse Model for Inherited Retinal Degeneration

**DOI:** 10.3390/ijms232314600

**Published:** 2022-11-23

**Authors:** Yujie Dong, Wenrong Xu, Yan Li, Chunling Wei, Yunzhang Hu, Zhulin Hu, François Paquet-Durand, Kangwei Jiao

**Affiliations:** 1 Yunnan Eye Institute & Key Laboratory of Yunnan Province, Yunnan Eye Disease Clinical Medical Center, Affiliated Hospital of Yunnan University, Yunnan University, 176 Qingnian, Kunming 650021, China; 2 Chinese Academy of Medical Sciences, Jiaoling, Kunming 650031, China; 3 Institute for Ophthalmic Research, Eberhard-Karls-Universität Tübingen, 72076 Tübingen, Germany

**Keywords:** retinitis pigmentosa, *PDE6* gene, MAPK/c-Jun-EGR1, photoreceptor cells, poly (ADP-ribose) polymerase-1

## Abstract

Retinitis pigmentosa (RP) is a group of inherited retinal dystrophies that typically results in photoreceptor cell death and vision loss. Here, we explored the effect of early growth response-1 (EGR1) expression on photoreceptor cell death in *Pde6b^rd1^* (*rd1*) mice and its mechanism of action. To this end, single-cell RNA-seq (scRNA-seq) was used to identify differentially expressed genes in *rd1* and congenic wild-type (WT) mice. Chromatin immunoprecipitation (ChIP), the dual-luciferase reporter gene assay, and western blotting were used to verify the relationship between EGR1 and poly (ADP-ribose) polymerase-1 (PARP1). Immunofluorescence staining was used to assess PARP1 expression after silencing or overexpression of EGR1. Photoreceptor cell death was assessed using the TUNEL assay following silencing/overexpression of EGR1 or administration of MAPK/c-Jun pathway inhibitors tanzisertib and PD98059. Our results showed differential expression of ERG1 in *rd1* and WT mice via scRNA-seq analysis. The ChIP assay demonstrated EGR1 binding to the *PARP1* promoter region. The dual-luciferase reporter gene assay and western blotting results revealed that EGR1 upregulated PARP1 expression. Additionally, the TUNEL assay showed that silencing EGR1 effectively reduced photoreceptor cell death. Similarly, the addition of tanzisertib and PD98059 reduced the expression of c-Jun and EGR1 and decreased photoreceptor cell death. Our study revealed that inhibition of the MAPK/c-Jun pathway reduced the expression of EGR1 and PARP1 and prevented photoreceptor cell death. These results highlight the importance of EGR1 for photoreceptor cell death and identify a new avenue for therapeutic interventions in RP.

## 1. Introduction

Retinitis pigmentosa (RP) is an inherited retinal disease characterized primarily by degenerative lesions in the photoreceptor cell layer [1]. The disease begins with the progressive loss of rod photoreceptor cells, followed by secondary degeneration and death of cone photoreceptors, ultimately leading to complete blindness in patients [2]. As photoreceptor cell death is the main cause leading to RP, studying the underlying mechanisms and rational design of appropriate interventions has the potential to alleviate the progression of the disease.

One of the best studied animal models for RP is the retinal degeneration-1 (*rd1*) mouse, which carries a loss of function mutation in the *Pde6b* gene [3]. This gene encodes the beta subunit of phosphodiesterase-6 (PDE6), an important regulator of the phototransduction cascade in retinal photoreceptor cells [4]. In rod photoreceptors, the PDE6 protein consists of two catalytic sub-units (α and β) and a regulatory sub-unit (γ), which are encoded by the *PDE6A*, *PDE6B*, and *PDE6G* genes, respectively [4,5]. Mutations occurring in any of the three *PDE6* subunit genes together may be responsible for up to 10% of human RP cases [6,7]. Mutations in *PDE6* genes can cause a sustained increase in the accumulation of cyclic guanosine monophosphate (cGMP) in the outer segments of rod photoreceptors, eventually causing these cells to die [6].

Early growth response 1 (*EGR1*), also known as *zif268* or *Tis8*, is an important member of the early growth response gene family [8]. Various extracellular stimuli activate *EGR1* to mediate the cellular stress response and act as transcription factors, with *EGR1* promoting the expression of other genes, as well as its own transcription [9]. Transcription of the *EGR1* gene is normally regulated by the mitogen-activated protein kinase (MAPK) signaling pathway and is primarily activated in response to two members: extracellular signal-regulated kinases 1/2 (ERK1/2) and c-Jun N-terminal kinases (JNK) [10,11]. The intracellular level of EGR1 is significantly increased after ERK1/2 and JNK activation [12]. Inhibiting the expression of EGR1 can reduce photoreceptor cell death [13,14], but the specific mechanism is unclear.

In previous studies, we observed increased levels of cGMP in *rd1* mice [15,16,17], while other investigators found c-Jun and JNK to be closely related to retinal cell death and early differentiation of photoreceptor cells [18,19]. Therefore, we hypothesized that cGMP induces photoreceptor cell death via the MAPK/c-Jun–EGR1 signaling cascade. In this study, we investigated expression levels of EGR1 in the *rd1* mouse model and whether inhibition of the MAPK/c-Jun pathway would positively influence EGR1 expression and photoreceptor cell death.

## 2. Results

**Figure 1:**
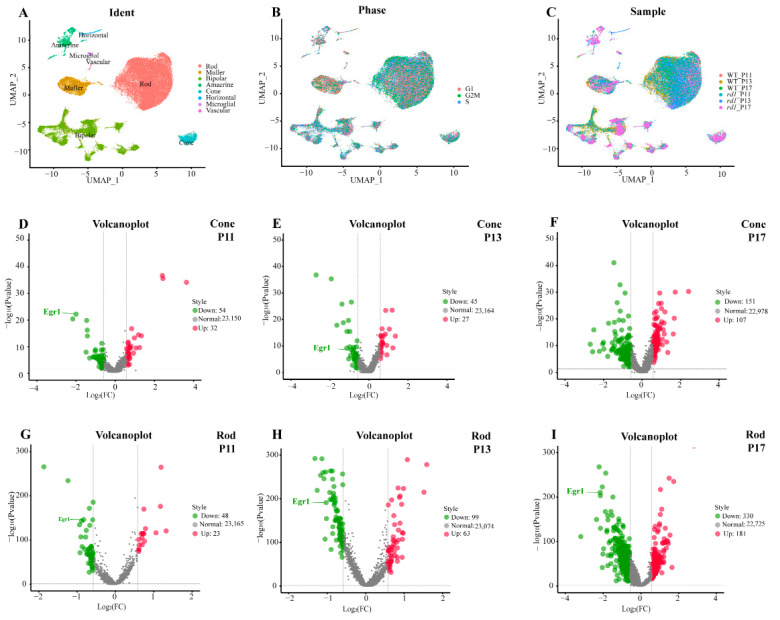
Single-cell RNA-seq analysis of wild-type and *rd1* rod and cone photoreceptors. (**A**) Distribution of cell clusters between wild-type (WT) and *rd1* mice. (**B**) Cell cycle of all cell clusters. (**C**) Cellular composition of sub-clusters in WT and *rd1* mice. (**D**–**I**) Volcano plots showing differentially expressed genes (|avg_logFC| > 0.58, *p* < 0.05) that were significantly downregulated (green) or upregulated (red) in rods and cones at P11, P13, and P17 in WT vs. *rd1* retinas. Note the prominent regulation of EGR1 in rod and cone photoreceptors.

Initially, we characterized the cellular composition of the retina and cell-type-specific gene expression patterns in wild-type (WT) and *rd1* mice using scRNA-seq analysis at post-natal (P) days 11, 13, and 17. After filtering out invalid cells, a total of 43,977 cells (5142 from WT P11, 12,737 from WT P13, 5189 from WT P17, 7748 from *rd1* P11, 7181 from *rd1* P13, and 5982 from *rd1* P17) were classified using unsupervised cell clustering analysis. The expression patterns of known marker genes of each retinal cell type were used to identify 20 different clusters, including rods, cones, bipolar cells, Müller cells, vascular cells, microglial cells, amacrine cells, and horizontal cells (Figure 1A). Cell cycle analysis indicated that rods and Müller cells may have greater capacity for proliferation than other cells (Figure 1B). The cellular composition of the photoreceptor sub-clusters in WT (P11, P13, P17) and *rd1* (P11, P13, P17) retinas was analyzed (Figure 1C). Two main cell clusters, rods and bipolar cells, were present in the retinas of WT and *rd1* mice, respectively. Corresponding volcano plots showed that EGR1 expression was downregulated in rods at P11, P13, and P17 and in cones at P11 and P13 in retinas of WT mice compared to those in the retinas of *rd1* mice (Figure 1D–I).

### 2.1. EGR1 Binds to the PARP1 Promoter Region

**Figure 2:**
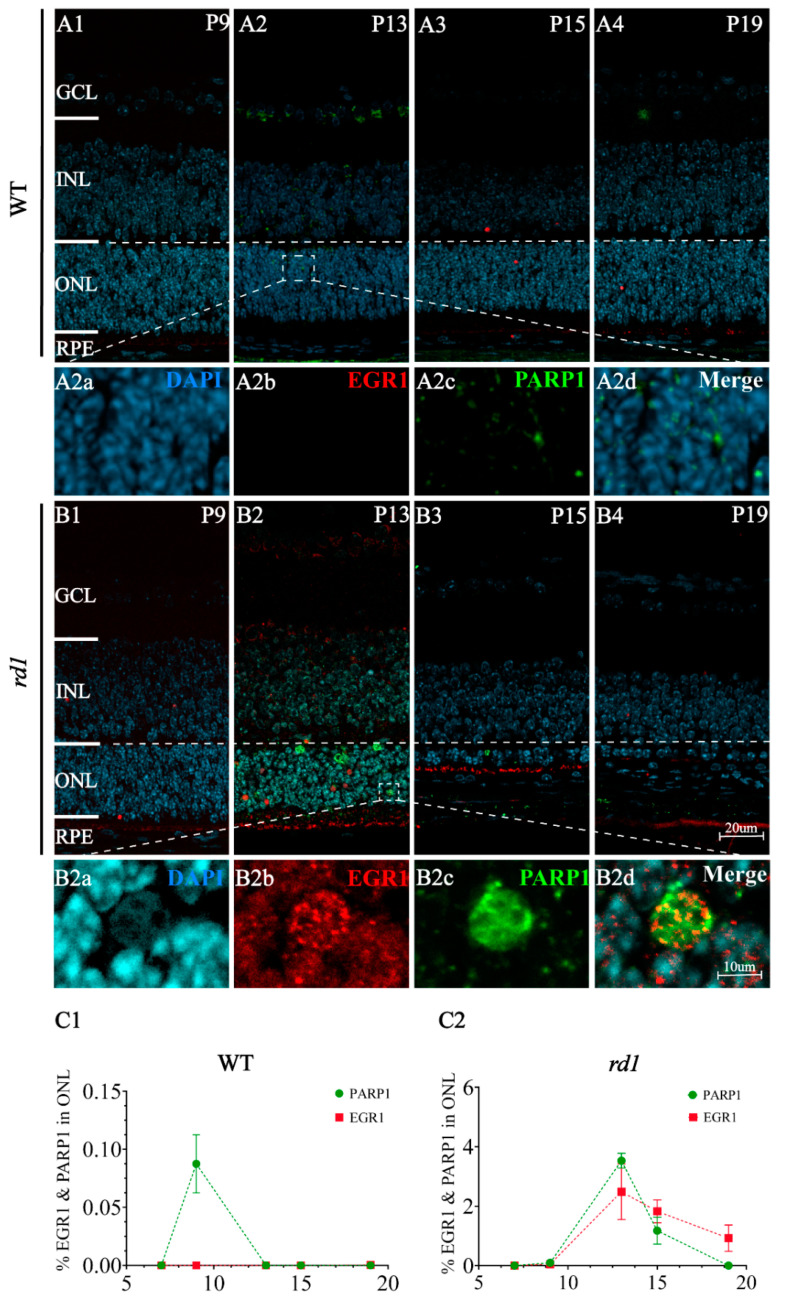
EGR1 and PARP1 spatially overlap in *rd1* mouse photoreceptors. (**A1**–**A4**,**B1**–**B4**) Immunofluorescence staining detected EGR1 (red) and PARP1 (green) expression at time points ranging from P9 to P19 in WT and *rd1* retinas in vivo. At P13, colocalization cells of EGR1 (**B2b**, red) and PARP1 (**B2c**, green) were detected in *rd1* (**B2**, **B2d** Merge), while little colocalized positive cells of EGR1 (**A2b**, red) and and PARP1 (**A2c**, green) were detected in WT (**A2**, **A2d** Merge). DAPI (4′,6-diamidino-2-phenylindole; light blue) was used as a nuclear counterstain (**A2a**,**B2a**). (**C1**,**C2**) Quantification of EGR1- and PARP1-positive cells in the outer nuclear layer (ONL) of WT and *rd1* retinas at P9, P13, P15, and P19. The images shown are representative of observations for at least six different specimens of each genotype. Error bars represent SD. INL = inner nuclear layer, GCL = ganglion cell layer, RPE = retinal pigment epithelium. Scale bar = 20 µm (10 µm in insert).

**Figure 3:**
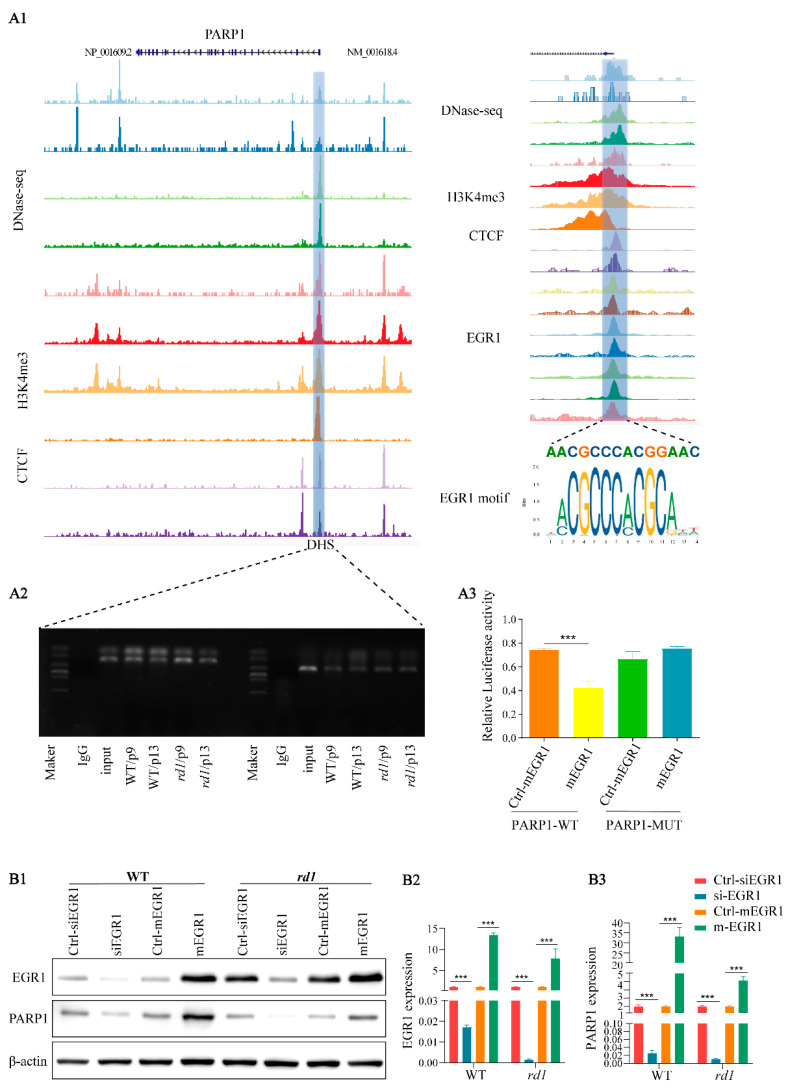
EGR1 binds to the *PARP1* promoter region. (**A1**) Left panel: Signal distribution in the vicinity of the *PARP1* locus via DNase-seq, ChIP-seq of H3K4me3, and CCCTC binding factor (CTCF) analysis of the human eye. Colored lines refer to a set of DNase /ATAC/ChIP-seq data, with peaks representing binding at this position. A DNA hypersensitive site (DHS) was found near the transcription start site (blue box). Right panel: Magnification of the *PARP1* promotor region and DHS site. An EGR1 binding motif was detected in the *PARP1* promoter region. (**A2**) DNA sequencing and ChIP-qPCR analysis revealed that EGR1 binds to the DHS in *PARP1*. (**A3**) The interaction between EGR1 and PARP1 was determined in 293T cell cultures by GLuc /SEAP dual luminescence assay. Co-transfection of EGR1 overexpression and PARP1 in the wild-type (WT) construct resulted in significant inhibition of luciferase activity, indicating an interaction between EGR1 and PARP1. However, such interaction was not observed in the *PARP1* mutant (MUT) construct, suggesting specific binding of EGR1 to the WT *PARP1* gene. (**B1**) EGR1 and PARP1 expression were analyzed by western blotting in *rd1* and WT retinal explants, cultured from P5 to P13, using either overexpression (mEGR1) or silencing of EGR1 (siEGR1). (**B2**,**B3**) Quantification of relative EGR1 and PARP1 protein expression. Error bars represent SD. *** = *p* < 0.001.

In our previous study, poly (ADP-ribose) polymerase-1 (PARP1) was shown to play a crucial role in photoreceptor cell death in *rd1* mice [20]. Immunofluorescence analysis showed that EGR1 and PARP1 expression spatially overlapped in retinal tissues of *rd1* mice (Figure 2). To further investigate the relationship between EGR1 and PARP1, DNA sequencing and chromatin immunoprecipitation (ChIP)-qPCR analysis was performed, revealing that EGR1 can bind to the Dnase hypersensitive site (DHS) in the promoter region of *PARP1* (Figure 3(A1,A2)). Moreover, the dual-luciferase reporter gene analysis also showed that EGR1 can bind to the *PARP1* promoter region (Figure 3(A3); Table S1). Additionally, western blotting showed that PARP1 expression was significantly decreased by EGR1 silencing but increased by EGR1 overexpression (Figure 3B). Together, these results suggested that EGR1 can regulate PARP1 expression.

### 2.2. Suppression of EGR1 Expression Inhibited Photoreceptor Cell Death

**Figure 4:**
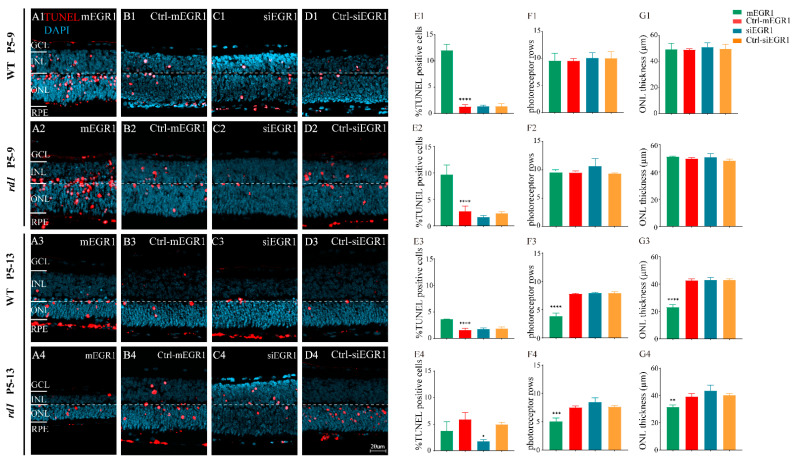
EGR1 expression promotes photoreceptor cell death in WT and *rd1* retinas. WT and *rd1* in vitro retinal explants cultured from P5 onwards were treated with different genetic constructs to either silence or overexpress EGR1. The TUNEL assay (red) was used to examine photoreceptor death at P9 and P13. DAPI (blue) was used as a nuclear counterstain. (**A1–A4**) Cross-sectional images of retinas treated with EGR1 overexpression vector (adeno-associated viral (AAV) vector, 1.84 × 10^10^ viral genomes (vg)/mL, 10 μL for each retinal explant). (**B1–B4**) Retinas treated with EGR1 control vector (negative control, AAV vector without mEGR1). (**C1–C4**) Retinas treated with siRNA construct for EGR1 knockdown (AAV9-Egr1-RNAi 1.57 × 10^9^ vg/mL, 10 μL for each retinal explant). (**D1–D4**) Retinas treated with control vector for EGR1 siRNA (negative control AAV vector, without siRNA EGR1). (**E1–E4,F1–F4,G1–G4**) Quantification of (**E1–E4**) TUNEL positive cells in the outer nuclear layer (ONL), (**F1–F4**) photoreceptor row counts, and (**G1–G4**) thickness of the ONL in µm. Images shown are representative of observations for at least six different specimens of each genotype. Error bars represent SD. ONL = outer nuclear layer, INL = inner nuclear layer, GCL = ganglion cell layer. Scale bar = 20 µm. * = *p* < 0.05; ** = *p* < 0.01; *** = *p* < 0.001; and **** = *p* < 0.0001.

**Figure 5:**
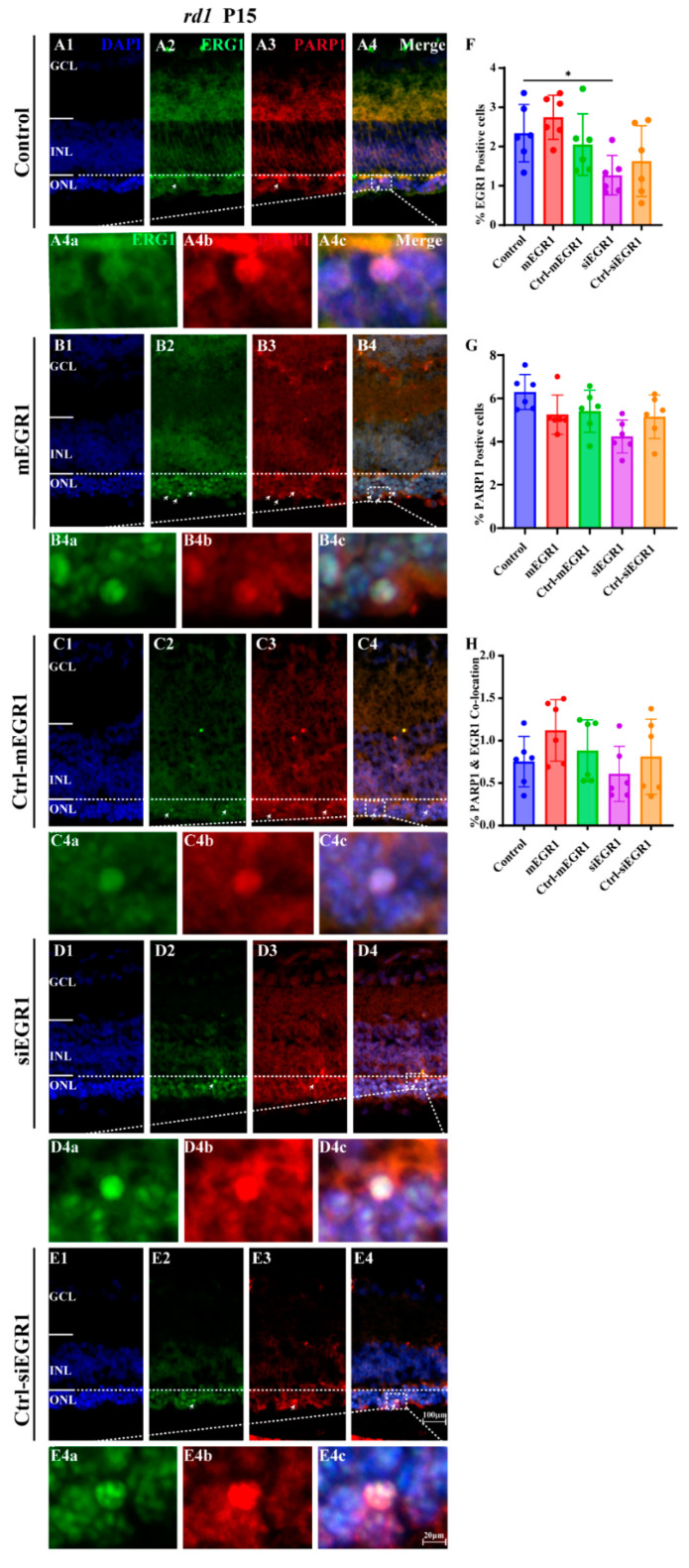
EGR1 and PARP1 expression colocalize in *rd1* photoreceptors. At P5, *rd1* mice were given a single suprachoroidal injection of AAV carrying constructs targeting EGR1. The expression of EGR1 (green) and PARP1 (red) was examined at P15 in *rd1* retinas using immunofluorescence staining. (**A1**–**A4**) Untreated, control *rd1* retina. (**B1**–**B4**) Retina after treatment with AAV9-EGR1-GFP (1.84 × 10^10^ vg/mL). (**C1**–**C4**) Retina after treatment with a control construct for EGR1. (**D1**–**D4**) *rd1* retina treated with siRNA construct AAV9-EGR1-RNAi (1.57 × 10^9^ vg/mL). (**E1**–**E4**) Retina after treatment with a control construct for siRNA EGR1. The arrows indicate the colocalization cells of EGR1 and PARP1 positive cells. The colocalized sections (merge, **A4c**,**B4c**,**C4c**,**D4c**,**E4c**) show the expression of EGR1 (green, **A4a**,**B4a**,**C4a**,**D4a**,**E4a**) and PARP1 (red, **A4b**,**B4b**,**C4b**,**D4b**,**E4b**), and nuclei were counterstained with DAPI (blue). (**F**–**H**) Quantification of (**F**) percentage of EGR1 positive cells in *rd1* outer nuclear layer (ONL), (**G**) percent PARP1-positive cells in ONL, (**H**) percentage of ONL cells displaying colocalization of EGR1 and PARP1 staining. Images shown are representative of observations for at least six different specimens of each genotype. Error bars represent SD. INL = inner nuclear layer, GCL = ganglion cell layer. Scale bar = 100 µm (20 µm in insert). * = *p* < 0.05.

**Figure 6:**
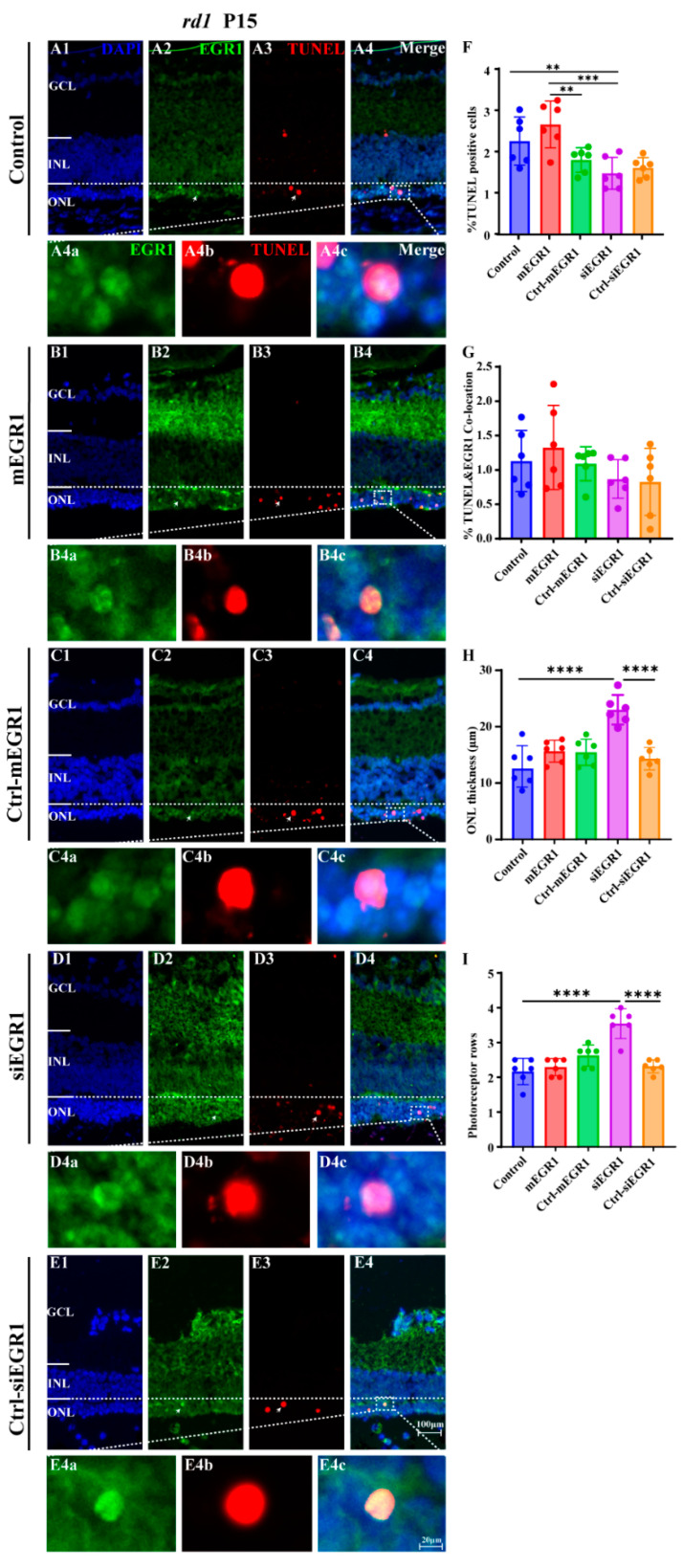
EGR1 expression is increased in dying *rd1* photoreceptors. The expression of EGR1 (green) and TUNEL (red) in the retinas of P15 *rd1* mice was examined using immunofluorescence staining and TUNEL assay. (**A1–A4**) Untreated, control *rd1* retina. (**B1–B4**) Retina after treatment with AAV9-EGR1-GFP (1.84 × 10^10^ vg/mL). (**C1–C4**) Retina after treatment with a control construct for EGR1. (**D1–D4**) *rd1* retina treated with siRNA construct AAV9-EGR1-RNAi (1.57 × 10^9^ vg/mL). (**E1–E4**) Retina after treatment with a control construct for siRNA EGR1. The arrows indicate the colocalization cells of EGR1 and TUNEL positive cells. The colocalized sections (merge, **A4c**,**B4c**,**C4c**,**D4c**,**E4c**) show the expression of EGR1 (green, **A4a**,**B4a**,**C4a**,**D4a**,**E4a**) and TUNEL positive cells (red, **A4b**,**B4b**,**C4b**,**D4b**,**E4b**), and nuclei were counterstained with DAPI (blue). (**F**–**I**) Quantification of (**F**) percentage of TUNEL-positive, dying cells in *rd1* outer nuclear layer (ONL), (**G**) percentage of ONL cells displaying colocalization of TUNEL and EGR1, (**H**) thickness of ONL in µm, and (**I**) photoreceptor row counts. Images shown are representative of observations for at least six different specimens of each genotype. The arrows indicate the colocalization cells of EGR1 and TUNEL. Error bars represent SD. INL = inner nuclear layer, GCL = ganglion cell layer. Scale bar = 100 µm (20 µm in insert). ** = *p* < 0.01; *** = *p* < 0.001; and **** = *p* < 0.0001.

To explore the effect of EGR1 expression on RP, we evaluated the number of TUNEL-positive cells in the outer nuclear layer (ONL) of retinal explants in vitro, cultured either from P5 to P9 or from P5 to P13, in WT and *rd1* mice after either silencing or overexpressing EGR1. Overexpression of EGR1 significantly promoted cell death in the ONL compared to that in the Ctrl-mEGR1 group (Figure 4(A1–A3, E1–E3)), except in *rd1* mice at P13 (Figure 4(A4, E4)). In addition, the number of photoreceptor rows and ONL thickness were sharply decreased by EGR1 overexpression at P13 (Figure 4(F3, F4, G3, G4)). Moreover, EGR1 silencing effectively reduced photoreceptor death at P13 in *rd1* mice (Figure 4(C4, E4)), with no effect on photoreceptor rows and ONL thickness (Figure 4(F4,G4)). Additionally, in vivo experiments revealed that overexpression of EGR1 upregulated the expression of PARP1 in *rd1* mice. Conversely, PARP1 expression was inhibited by EGR1 silencing (Figure 5). Furthermore, immunofluorescence indicated increased expression of EGR1 in dying *rd1* photoreceptors (Figure 6). However, in WT retinas, the expression of EGR1, PARP1, and amount of cell death observed (TUNEL-positive cells) were extremely low (Supplementary Figures S3 and S4).

### 2.3. Inhibition of MAPK/c-Jun Pathway Activation Suppressed Photoreceptor Cell Death

**Figure 7:**
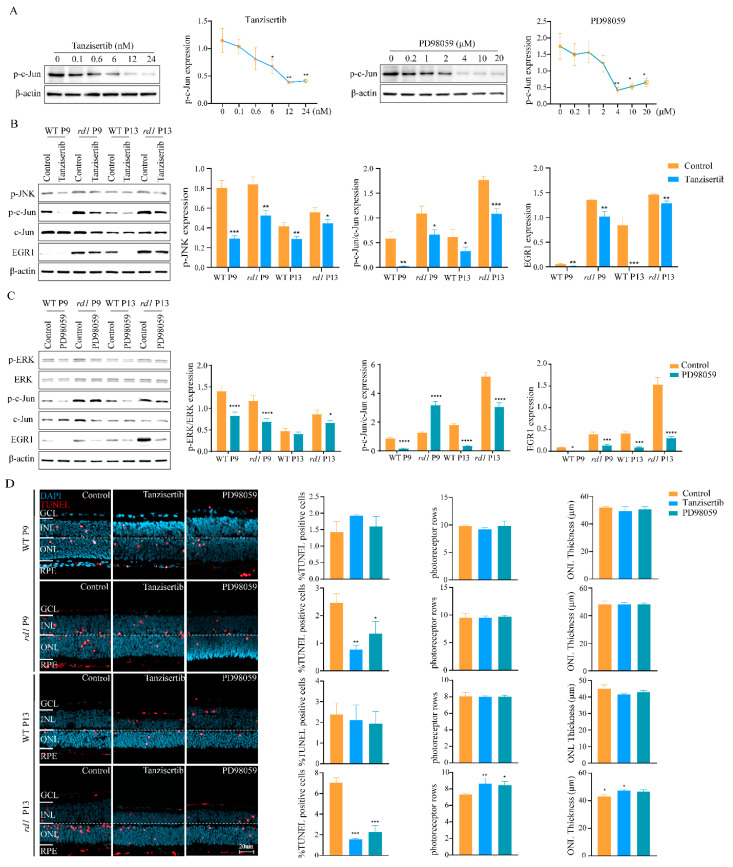
Inhibition of the MAPK/c-Jun pathway suppresses photoreceptor cell death. (**A**) Western blotting was used to analyze the effects of tanzisertib (JNK inhibitor) and PD98059 (ERK inhibitor) on in vitro retinal explants cultured from P5 to P9 or from P5 to P13 to analyze c-Jun phosphorylation in response to different inhibitor concentrations. (**B**) The expression of EGR1, p-c-Jun, and p-JNK was analyzed using western blotting after tanzisertib treatment. Compared to the control, tanzisertib reduced p-JNK and p-c-Jun/c-Jun expression in WT and *rd1* retinas and was accompanied by downregulation of EGR1 expression (*p* < 0.05). (**C**) The expression of EGR1, p-c-Jun, and p-ERK was analyzed by western blotting after PD98059 addition. (**D**) After treatment with tanzisertib and PD98059, the TUNEL assay (red) was used to quantify the numbers of dying cells in the outer nuclear layer (ONL). DAPI (blue) was used as nuclear counterstain. The percentages of TUNEL-positive cells, photoreceptor row count, and ONL thickness (µm) were quantified in WT and *rd1* mice at P9 and P13. Images shown are representative of observations for at least six different specimens of each genotype. Error bars represent SD. INL = inner nuclear layer, GCL = ganglion cell layer. Scale bar = 20 µm. * = *p* < 0.05; ** = *p* < 0.01; *** = *p* < 0.001; and **** = *p* < 0.0001.

Since activation of the MAPK/c-Jun pathway can promote the expression of EGR1 [21,22], we examined EGR1 expression and photoreceptor death after administration of the JNK inhibitor tanzisertib and ERK inhibitor PD98059. Western blotting and the TUNEL assay were used as readouts. Tanzisertib and PD98059 reduced c-Jun phosphorylation levels, with optimal working concentrations of 12 nM and 4 μM, respectively (Figure 7A). Administration of PD98059 significantly reduced the expression of EGR1, p-c-Jun, and p-ERK. Similarly, tanzisertib treatment significantly reduced (*p* < 0.05) the expression of EGR1, p-c-Jun, and p-JNK (Figure 7B,C).

We then tested whether the two drugs could reduce photoreceptor cell death in retinal explant cultures derived from P5 WT and *rd1* animals. These explant cultures were ended at either P9, a time-point before the onset of widespread *rd1* degeneration, and at P13, the peak of *rd1* photoreceptor cell death. In WT retinal explants, tanzisertib and PD98059 showed neither positive nor negative effects on photoreceptor cell death. In *rd1* retinas, however, both drugs already reduced photoreceptor cell death at P9, indicating that EGR1 signaling had an early detrimental effect on photoreceptor viability. Importantly, at P13, the addition of tanzisertib and PD98059 not only reduced the number of dying cells as evidenced by the TUNEL assay, but also increased the number of photoreceptor rows and ONL thickness (Figure 7D).

## 3. Discussion

A characteristic pathological feature of RP is the loss of photoreceptor cells in the ONL [23,24], yet the precise mechanisms triggering photoreceptor degeneration and death remain poorly understood. In this study, we showed that EGR1 expression was positively correlated with the expression of PARP1 and photoreceptor cell death in the *rd1* mouse model for RP. Moreover, we found that inhibition of the MAPK/c-Jun pathway reduced EGR1 and PARP1 expression and ultimately prevented photoreceptor cell death.

In the outer segments of photoreceptors, the second messenger cGMP regulates the influx of Na^+^ and Ca^2+^ ions via activation of the cyclic-nucleotide-gated (CNG) channel [16]. In addition, cGMP activates cGMP-dependent protein kinase (protein kinase G; PKG) [15,25]. Both cGMP targets may promote photoreceptor cell death, either via an excessive influx of Ca^2+^ [26] or via increased phosphorylation of PKG target proteins [27]. Many different RP disease-related genes have been connected to excessive accumulation of cGMP in photoreceptors [28,29], which may thus constitute a common signal that can trigger photoreceptor cell death [16]. Accordingly, many previous studies have demonstrated that increased cGMP levels in rod photoreceptors lead to progressive photoreceptor degeneration [15,30,31]. Notably, the cGMP-dependent activation of PKG may entail the activation of c-Jun [32].

ERK1/2 and JNK are activated by phosphorylation under external stimuli, thereby increasing the expression level of intracellular EGR1 [12]. c-Jun is activated by the phosphorylation of serine residues 63 and 73 by JNK [33], and can then transfer to the nucleus to bind to EGR1 and eventually cause cell death [12,34]. Our results demonstrated that the administration of PD98059 and tanzisertib reduced the expression of ERK1/2, JNK, c-Jun, and EGR1. Importantly, PD98059 and tanzisertib addition inhibited the cell death of photoreceptor cells. These results suggest that inhibition of the MAPK/c-Jun-EGR1 signaling cascade may suppress photoreceptor cell death.

EGR1 is associated with cell death in photoreceptor cells [13,14], but the detailed mechanism of action is not clear. EGR1 presumably binds to the promoter region of genes to promote transcription of downstream target genes, including growth factors, growth factor receptors, extracellular matrix proteins, and others, thereby participating in the regulation of cell growth, differentiation, proliferation, and cell death [35,36]. In this study, DNA sequencing and ChIP assay results revealed that EGR1 could bind to the *PARP1* promotor region, which was further confirmed via the dual-luciferase reporter gene assay and western blot experiments, indicating that EGR1 can upregulate PARP1 expression. In addition, the silencing of EGR1 expression reduced PARP1 expression and photoreceptor cell death. Since PARP1 is known to promote photoreceptor cell death in a variety of RP animal models [20,37,38], we speculate that EGR1 controls photoreceptor cell death via regulation of PARP1 expression.

**Figure 8:**
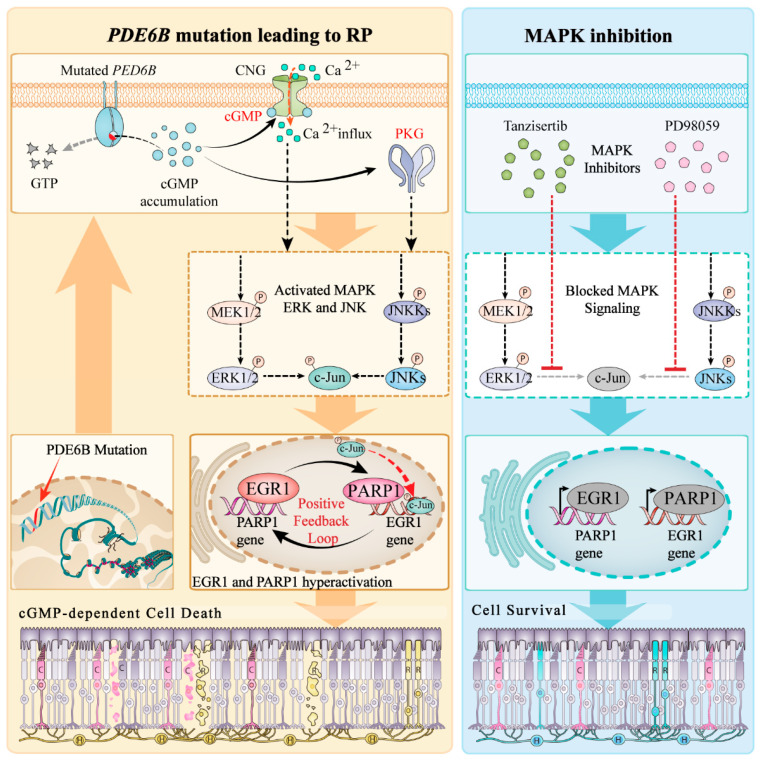
Role of EGR1 and MAPK signaling in photoreceptor cell death. (**Left**) Disease-causing mutations in the *PDE6B* gene induce photoreceptor cGMP accumulation. This in turn activates cyclic nucleotide gated (CNG) channels in the outer segment, leading to Na^+^-and Ca^2+^-influx, photoreceptor depolarization, and parallel activation of protein kinase G (PKG). Further downstream, MAPK, ERK, and JNK signaling pathways are activated, likely causing c-Jun to translocate to the nucleus and promoting EGR1 expression. This may drive a positive feedback loop with EGR1 and PAPR1 as core molecules, expressing a large amount of EGR1 protein to bind to the *PARP1* gene promoter region, further stimulating its transcription and causing photoreceptor cell death. (**Right**) Inhibition of c-Jun nuclear translocation with either tanzisertib or PD98059 can reduce the expression of EGR1 and PARP1, thereby delaying cell death.

Based on the above experimental results, we propose the following roles for EGR1 and MAPK signaling in photoreceptor cell death (Figure 8): a *PDE6B*-mutation induces increased photoreceptor cGMP levels, which causes an overactivation of both the CNG-channel and PKG, leading to an influx of Na^+^ and Ca^2+^ and photoreceptor depolarization on the one hand, while activating the MAPK/c-Jun signaling pathway on the other hand. At present, it is not entirely clear whether activation of these pathways is brought about by direct PKG-dependent phosphorylation, Ca^2+^ signaling, or a combination of both [39]. Whatever the case, the downstream activation of c-Jun and its nuclear translocation is connected to increased EGR1 and PARP1 expression. Notably, overactivation of PARP1 may precipitate cell death via either excessive consumption of its substrate NAD^+^ or aberrant ADP-ribose signaling [40].

While our study sheds light on the role of EGR1 signaling in photoreceptor degeneration, the study has several limitations. Firstly, our results showed that targeting EGR1 can be effective in reducing photoreceptor death/loss, but it may also negatively regulate oxidative phosphorylation and mitochondrial pathways, effects which could potentially cause retinal degeneration via oxidative stress, cell cycle arrest, and/or DNA damage. Therefore, inhibition or downregulation of EGR1 may have unintended detrimental side effects and might not ensure the rescue of photoreceptor functionality. To address this point, future research may focus on post-intervention functional evaluation of the retina using, for instance, micro-electrode-array (MEA) and in vitro μERG recordings on retinal explants [39] or in vivo ERG on live treated animals [41].

An important hurdle that needs to be overcome before in vivo testing can be successfully conducted concerns drug administration and delivery. Since systemic exposure to drugs such as tanzisertib or PD98059 and general inhibition of MAPK/c-Jun/EGR1-signaling are likely to cause detrimental side effects, a drug delivery vehicle should be designed that will enable sustained drug release and retinal delivery after local administration, for instance, via intravitreal injection [42]. While this will require significant development work, local drug delivery will dramatically reduce systemic exposure and may be essential for facilitating long-term tolerability in chronic retinal diseases such as RP.

## 4. Materials and Methods

Animal models. We used C3H *Pde6b^rd/rd1^* mice as well as congenic C3H *Pde6b^+/+^* mice (hereafter, termed “*rd1*” and “WT”, respectively), irrespective of gender. These animals were originally provided by the Cell Death Mechanism group, Institute for Ophthalmic Research, Tübingen University (Germany) and were purposely bred for the present study. Both animal colonies were regularly genotyped to ensure that they indeed carried (or not) the disease-causing mutations. All mice were housed under a 12 h light/dark cycle with free access to food and water. All procedures were performed in compliance with the ARVO statement for the use of animals in Ophthalmic and Visual Research. Protocols were reviewed and approved by the ethical review board of the Affiliated Hospital of Yunnan University (No. 20180331).

Single-cell RNA-seq (scRNA-seq) and bioinformatics analysis. Retinal single-cell suspensions were prepared according to methods described in a previous study [43]. *rd1* and WT mice were sacrificed regardless of gender at different time points of P11 (*n* = 3; retinas *n* = 6), P13 (*n* = 3; retinas *n* = 6) and P17 (*n* = 3; retinas *n* = 6). The eyeballs were quickly placed into DPBS (phosphatide-buffered saline without calcium and magnesium CAT: 21-040-CVC, CORNING) which was pre-cooled at 4 °C in order to remove blood and impurities, incubated in 0.12% Proteinase K (Millipore, 539480) at 37 °C for 1 min and basal medium (Gibco, C11875500BT) with 50% fetal bovine serum (Gemini, 900-108) for 2 min, and then transferred to fresh DPBS for a final wash. After the cornea, sclera, iris, lens, and vitreous were removed on ice under the microscope, the retinal tissues containing retina-RPE-choroid were completely immersed in MACS Tissue Storage Solution (Miltenyi, 130-100-008), which was pre-cooled at 4 °C, and analyzed immediately to ensure that the activity and numbers of retinal cells were sufficient for further experimental analysis. Libraries were prepared using the Chromium Single Cell 3′ Reagent Kit v3 (10× Genomics (Shanghai) Co., Ltd., Shanghai, China) and sequenced on an Illumina NovaSeq PE150 instrument. Retinal scRNA-seq analyses were performed using the Seurat package in R [44]. Briefly, cells with a significant number of outlier genes (potential polysomes) and high percentage of mitochondrial genes (potential dead cells) were excluded from using the “FilterCells” function. The LogNormalize method was used to normalize gene expression. Principal component analysis (PCA) was then performed to reduce the dimensionality of the dataset using *t*-SNE/UMAP dimensionality reduction. Seurat was used to cluster cells based on the PCA scores. For every single cluster, differentially expressed genes (DEGs) were identified using the “FindAllMarkers” function in the Seurat package, and the screening threshold was set to |avg_logFC| > 0.58 and *p* < 0.05.

Retinal explant cultures and transfection. Retinal explant cultures (from P5-P19 and P5-P13) were prepared according to previously described methods [20]. siRNA EGR1 adeno-associated virus (AAV) vector (siEGR1) (1.57 × 10^9^ vg/mL, 10 µL for each retinal explant), negative control AAV vector without siRNA EGR1 (Ctrl-siEGR1) (5.5 × 10^9^ vg/mL, 10 µL for each retinal explant), overexpression m-EGR1 AAV vector (mEGR1) (1.84 × 10^10^ vg/mL, 10 µL for each retinal explant), and negative control AAV vector without m-EGR1 (Ctrl-mEGR1) (5.5 × 10^9^ vg/mL, 10 µL for each retinal explant) were used for transfection of retinal explants using the HighGene transfection reagent (Genechem, Shanghai, China) following the manufacturer’s instructions. In brief, the AAV9-siEGR1 vector was based on GV478, with the element order: U6-MCS-CAG-EGFP. The AAV9-mEGR1 (NM_007913) vector was based on GV590, element order: rpe65p-MCS-EGFP-3Flag-SV40 PolyA, Cloning site: NcoI / NcoI.

Suprachoroidal injection of vector in mice. After anesthesia with ketamine (100 mg/kg) by intraperitoneal injection, postnatal day 5 (P5) mice were suprachoroidally injected with siRNA EGR1 AAV vector (siEGR1) (1.57 × 10^9^ vg/mL), negative control AAV vector without siRNA EGR1 (Ctrl-siEGR1) (5.5 × 10^9^ vg/mL), overexpression m-EGR1 AAV vector (mEGR1) (1.84 × 10^10^ vg/mL) and negative control AAV vector without m-EGR1 (Ctrl-mEGR1) (5.59× 10^9^ vg/mL) (Genechem, Shanghai, China). Viral solutions were diluted in complete medium (CM). For controls, animals were injected with CM without AAV vector. A 40-gauge needle on a 5 µL Hamilton syringe (Hamilton Company, Reno, NV) was used to generate a small scleral tunnel incision to the limbus, and the vector was inserted into the scleral tunnel with the bevel facing downward and slowly advanced through the remaining scleral fibers into the suprachoroidal space. The mice were sacrificed at P15 and their eyeballs were removed for subsequent experiments. Visualization of the fundus showed a shallow choroidal detachment on the side of the injection site (Supplementary Figure S1). Expression of GFP was observed in retina and retinal pigmented epithelium after suprachoroidal injection of AAV9-EGR1 (Supplementary Figure S2).

Immunofluorescence. Frozen sections were washed with PBS for 15 min, and then incubated with EGR1 (1:500, Invitrogen, CAT: MA5-15009, RRID: AB_10982091, Thermo Fisher, China) and PARP1 (1:4000, Servicebio, CAT: GB111501, China) antibodies at 4 °C overnight. The following day, the sections were incubated with secondary antibody (FITC/Cy3-labelled) for 1 h in the dark. After washing with PBS, the sections were stained with 4′,6-diamidino-2-phenylindole (DAPI; Servicebio, Wuhan, China) in the dark for 10 min, followed by the addition of a spontaneous fluorescence quencher (Servicebio) for 5 min. Light and fluorescence microscopy was performed with a Zeiss Imager M2 Microscope equipped with a Zeiss Axiocam digital camera (Zeiss, Oberkochen, Germany). Images were captured using Zeiss Axiovision 4.7 software, and representative pictures were obtained from central areas of the retina.

TUNEL assay. Retinal tissue sections were prepared according to a previously reported method [20] and cell death was detected using the TUNEL kit (In Situ Cell Death Detection Kit, TMR red, 12156792910, ROCHE, Switzerland), according to the manufacturer’s protocol. Briefly, sections were incubated with TUNEL solution at 37 °C for 1 h in the dark and with DAPI at room temperature for 5 min in the dark. Subsequently, the number of TUNEL-positive cells was determined using a fluorescence microscope (Olympus, Japan). For cell quantification, whole radial slice pictures were captured using the Mosaix mode of Axiovision 4.7.

Quantitative real-time PCR (qRT-PCR) analysis. Total RNA was extracted from retinal tissues and cells using TRIzol reagent (QIAGEN, Dusseldorf, Germany) and reverse-transcribed into cDNA using a PrimeScript™ II 1st Strand cDNA synthesis kit (TaKaRa, Kyoto, Japan). qRT-PCR was performed using the Biosystems 7300 real-time PCR system. The thermocycling conditions were as follows: 95 °C for 1 min, followed by 40 cycles of 95 °C for 15 s, and 60 °C for 1 min. GAPDH served as an internal control, and the relative expression of genes was calculated using the 2-∆∆Ct method. The primer sequences were as follows: EGR1, forward 5′-CCA TTT AAG ACA GAA GGA CAA GAA-3′ and reverse 5′-GTA AGA GAG TGA AGA GGC AGC-3′; GAPDH, forward 5′-CTT TGG CAT TGT GGA AGG GCT C-3′ and reverse 5′-GCA GGG ATG TTC TGG GCA G-3′.

Western blot. Total protein was extracted from retinal tissues and cells using RIPA lysis buffer (Beyotime, Shanghai, China). Protein samples were separated on 10% polyacrylamide gels containing 0.1% SDS and transferred to polyvinylidene fluoride membranes. The membranes were blocked with 5% bovine serum albumin for 1 h and then incubated with primary antibody (1:1000, Beyotime, China) in blocking buffer at 4 °C overnight. The following day, the membranes were incubated with goat anti-rabbit IgG (H + L) secondary antibody labeled with horseradish peroxidase (HRP) (1:1000, Beyotime, China) for 1 h at room temperature. The bands were visualized using an ECL Plus Detection System (CAT: WBKLS0100, Immobilon Western HRP, Millipore, Germany).

Chromatin immunoprecipitation (ChIP) and ChIP-qPCR analysis. ChIP analysis was performed using a ChIP kit (Cell Signaling Technology, Danvers, MA, USA) according to the manufacturer’s instructions. After DNA purification, part of the sample was used for sequencing analysis, and the rest was used for PCR analysis. The resulting DNA was analyzed using qPCR and normalized to total chromatin (input). The sequences of the two primer pairs for *PARP1* were as follows: *PARP1-1*, forward 5′-AGG CAC CCG CAA CCC GC-3′ and reverse 5′-GGC CCG CAC CTG CAC CA-3′; *PARP1-2*, forward 5′-GGG AGG GGT TGG GGG TAA A-3′ and reverse 5′-AGC GAG TCC TTG GGG ATG C-3′.

Secrete-Pair™ Dual Luminescence Assay. The activities of Gaussia luciferase (GLuc) and secreted alkaline phosphatase (SEAP) in the dual luminescence reporting system were detected (Table S1). The luciferase reporter plasmid was built using the pmirGLO vector, into which the wild type and mutant type candidate genes were cloned. For this purpose, an appropriate amount of 293T cells were seeded into a 12-well plate (0.1 × 10^6^/cm^2^) and cultured at 37 °C in an incubator overnight. The PARP1-WT (vector inserting the wild-type *PARP1* promoter sequence) and PARP1-MUT (vector inserting an inverted sequence of the *PARP1* promoter) fragments were subcloned into the luciferase gene CS-HPRM43771-PL01 vector (Promega, Madison, WI, USA) to construct PARP1 (WT) and PARP1 (MUT) plasmids, respectively. Four groups were involved, including overexpressing EGR1 combined with PARP1-WT (mEGR1 + PARP1-WT), blank control with PARP1-WT (Ctrl-mEGR1 + PARP1-WT), overexpression of EGR1 combined with PARP1-WT (mEGR1 + PARP1-MUT) and blank control with PARP1-MUT (Ctrl-mEGR1 + PARP1-MUT). Luciferase reporter plasmids and regulating factors were co-transfected into 293T cells (Tsingke, Beijing, China) using Lipofectamine^®^ 3000 reagent (Thermo Fisher Scientific, Waltham, MA, USA). After 24 h, the cells were analyzed using the Secrete-Pair™ Dual Luminescence Assay kit (GeneCopoeia, LF033, Rockville, MD, USA), and luciferase activity was assessed using a luminescence plate reader (Molecular Devices Inc., Sunnyvale, USA).

Statistical analysis. Labelled cells were counted manually. The total number of cells was determined by dividing the outer nuclear layer (ONL) area by the average cell size. The total number of ONL cells was determined by dividing the percentage of positive cells by the number of positive cells. Three sections from at least three different animals were tested for each genotype and experimental condition. Statistical comparisons between experimental groups were performed using one-way ANOVA and Bonferroni’s correction using Prism 8 software for Mac OS (Graph Pad Software, La Jolla, CA, USA). Values are presented as the mean ± standard deviation (SD). Levels of significance were as follows: * = *p* < 0.05; ** = *p* < 0.01; *** = *p* < 0.001; and **** = *p* < 0.0001.

## 5. Conclusions

In summary, we found that EGR1 expression was upregulated in *rd1* mice. Silencing of EGR1 or administration of MAPK/c-Jun pathway inhibitors downregulated the expression of EGR1 and suppressed photoreceptor cell death. Additionally, we found that EGR1 could bind to the promotor region of *PARP1* and upregulate its expression. Therefore, we can conclude that mutations in the *PDE6B* gene lead to cGMP accumulation, causing activation of cGMP-PKG signaling and, further downstream, activation of the MAPK/c-Jun signaling pathway. This, in turn increases expression of EGR1 and PARP1, likely causing photoreceptor cell death. The present work has important implications for future studies investigating retinal function and neuroprotection. Notably, drugs targeting cGMP-signaling and/or the MAPK/c-Jun pathway may have therapeutic potential for the treatment of RP and related retinal diseases.

## Data Availability

The series entry (GSE212183, https://www.ncbi.nlm.nih.gov/geo/query/acc.cgi?acc=GSE212183 (accessed on 19 October 2022)) provides access to all of our data and is the accession that can be quoted in any article discussing the data.

## Supplementary Materials

The following supporting information can be downloaded at: https://www.mdpi.com/article/10.3390/ijms232314600/s1.
