# Supplementary material for: Inhibition of the MAPK/c-Jun-EGR1 Pathway Decreases Photoreceptor Cell Death in the rd1 Mouse Model for Inherited Retinal Degeneration
Source: Int J Mol Sci. 2022 Nov 23;23(23):14600. doi: 10.3390/ijms232314600 (PMC9740268; doi:10.3390/ijms232314600)
Supplement: Supplementary file 1 [file ijms-23-14600-s001.zip › ijms-1916993-supplementary.pdf]

International Journal of
Molecular Sciences

MDPI

Supplementary data and figures

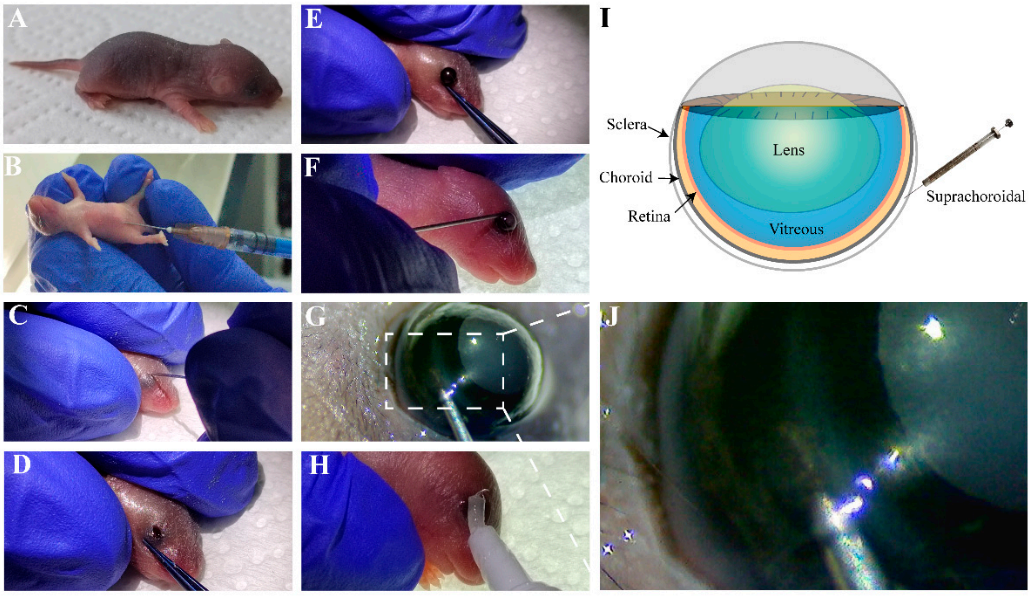

Figure S1. Suprachoroidal injection of vector in mice. Suprachoroidal injection of AAV vector in mice. (A) Mice at postnatal day 5. (B) Ketamine administration (100 mg/kg) for intraperitoneal anesthesia. (C-E) Use of a 1.5 mm incision of the eyelid with a 1 ml syringe needle for eyelid separation, to expose the eyeball. (F, G) A 34G Hamilton syringe was used for suprachoroidal injection of 2 μl AAV virus vector (AAV9-EGR1-GFP $1.84{\times}10^{10}$vg/ml, AAV9-EGR1-RNAi$1.57{\times}10^{9}$vg/ml). (H) The eyeball is pushed back and antibiotic eye ointment is applied. (I) Diagram illustrating suprachoroidal injection. (J) magnified view of needle inserted into the eye ball.

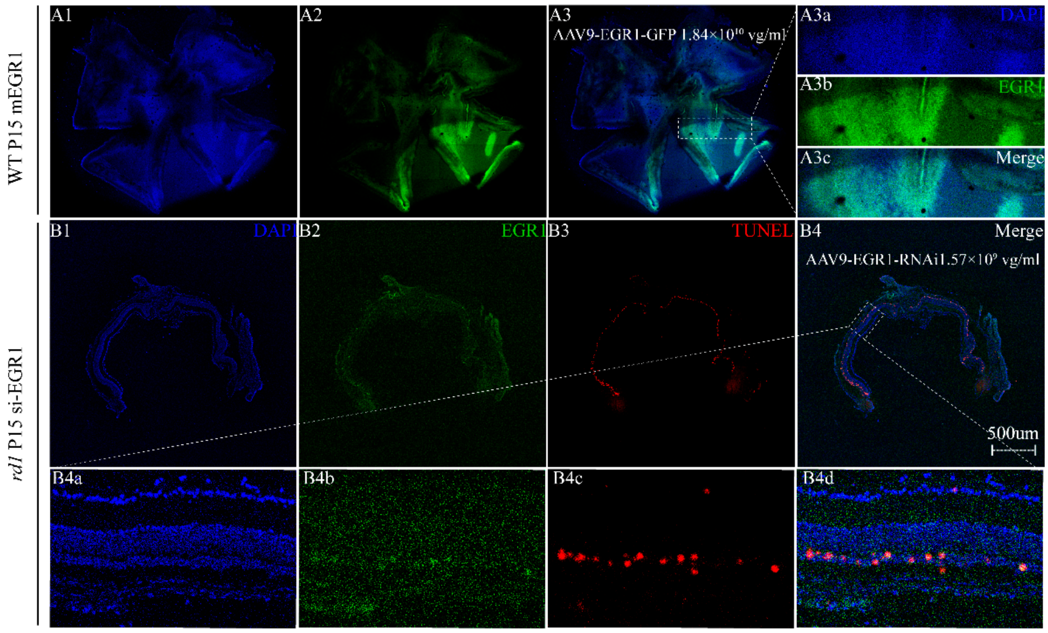

Figure S2. Expression of GFP in the retina after suprachoroidal injection of AAV9- EGR1-GFP. Wild-type (WT) and *rd1* mice were given a suprachoroidal injection of AAV9-EGR1-GFP $1.84{\times}10^{10}$ viral genomes (vg) / ml or AAV9-EGR1-RNAi$1.57{\times}10^{9}$ vg / ml. After 10 days, retinal flat mounts or ocular sections were examined by fluorescence microscopy. (A1-A3)

10 days after injection of AAV9-EGR1-GFP, retinal flat mounts (scale bar = 500μm) showed a few focal areas of hyperfluorescence around the injection site, but nothing above background elsewhere in the retina or RPE. An ocular section near the injection site (Merge, A3c) was examined for GFP fluorescence (green, A3b) and nuclei were counterstained with DAPI (blue, A3a, scale bar=500μm). An ocular section through the center of the cornea showed GFP fluorescence in photoreceptors (B1-B4, scale bar=500μm). The section (B4d, Merge) was immunohistochemically stained with GFP (green, B4b) and immunohistochemically stained for TUNEL (red, B4c), and nuclei were counterstained with DAPI (blue, B4a), which showed strong expression of GFP in photoreceptor outer segments and nuclei.

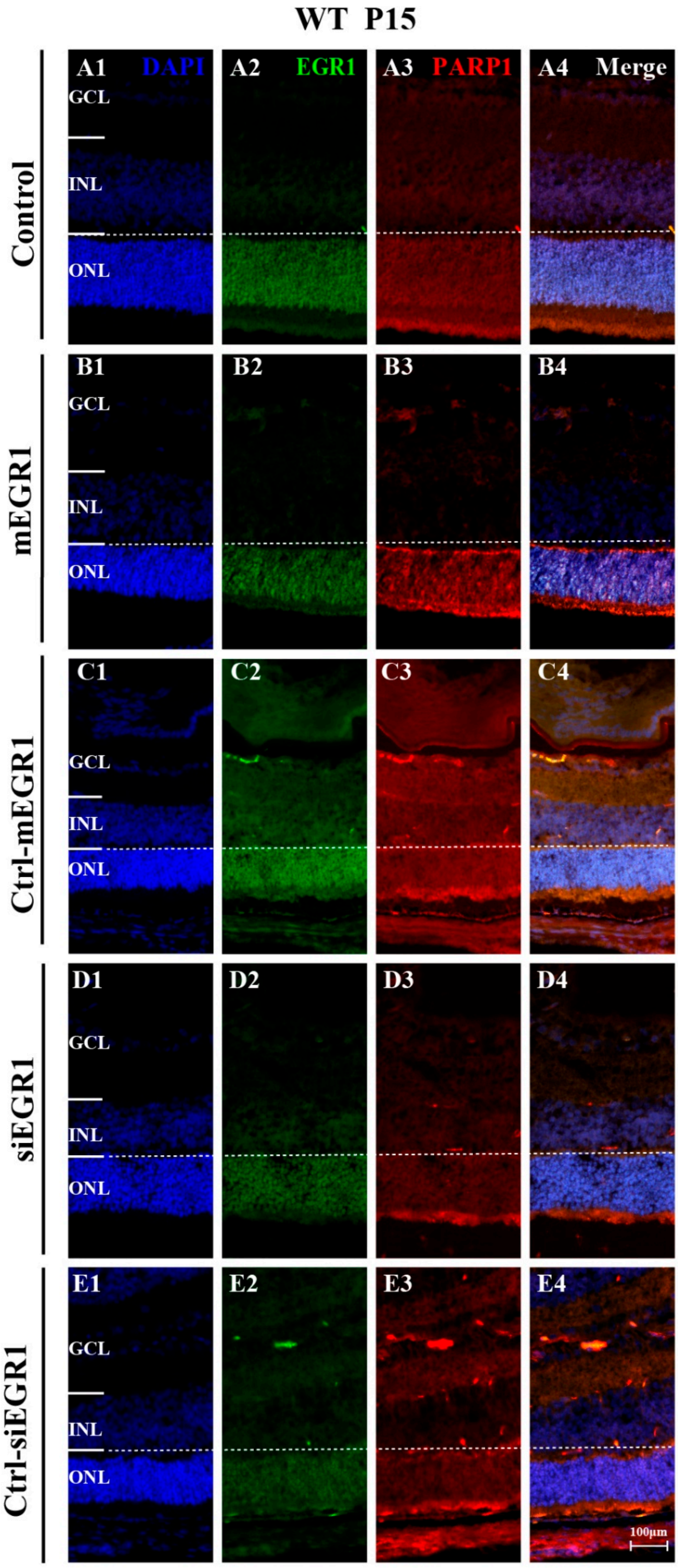

Figure S3. EGR1 and PARP1 expression are low in wild-type retina. At P5, wild-type (WT) mice were given a single suprachoroidal injection of an AAV carrying constructs targeting EGR1. The expression of EGR1 (green) and PARP1 (red) was examined at P15 in WT retina using immunofluorescence staining. Nuclei were counterstained with DAPI (blue). (A-E) In WT retina there were only few cells positive for EGR1 and PARP1 expression. (A1-A4) Untreated, control

rd1 retina. (B1-B4) Retina after treatment with AAV9-EGR1-GFP (7.68×10^{10} vg/ml). (C1-C4) Retina after treatment with a control construct for EGR1. (D1-D4) *rd1* retina treated with siRNA construct AAV9-EGR1-RNAi (3.676×10^{9} vg/ml). (E1-E4) Retina after treatment with a control construct for siRNA EGR1. The images shown are representative for observations on at least six different specimens for each genotype. ONL = outer nuclear layer, INL = inner nuclear layer, GCL = ganglion cell layer. Scale bar = 100 μm.

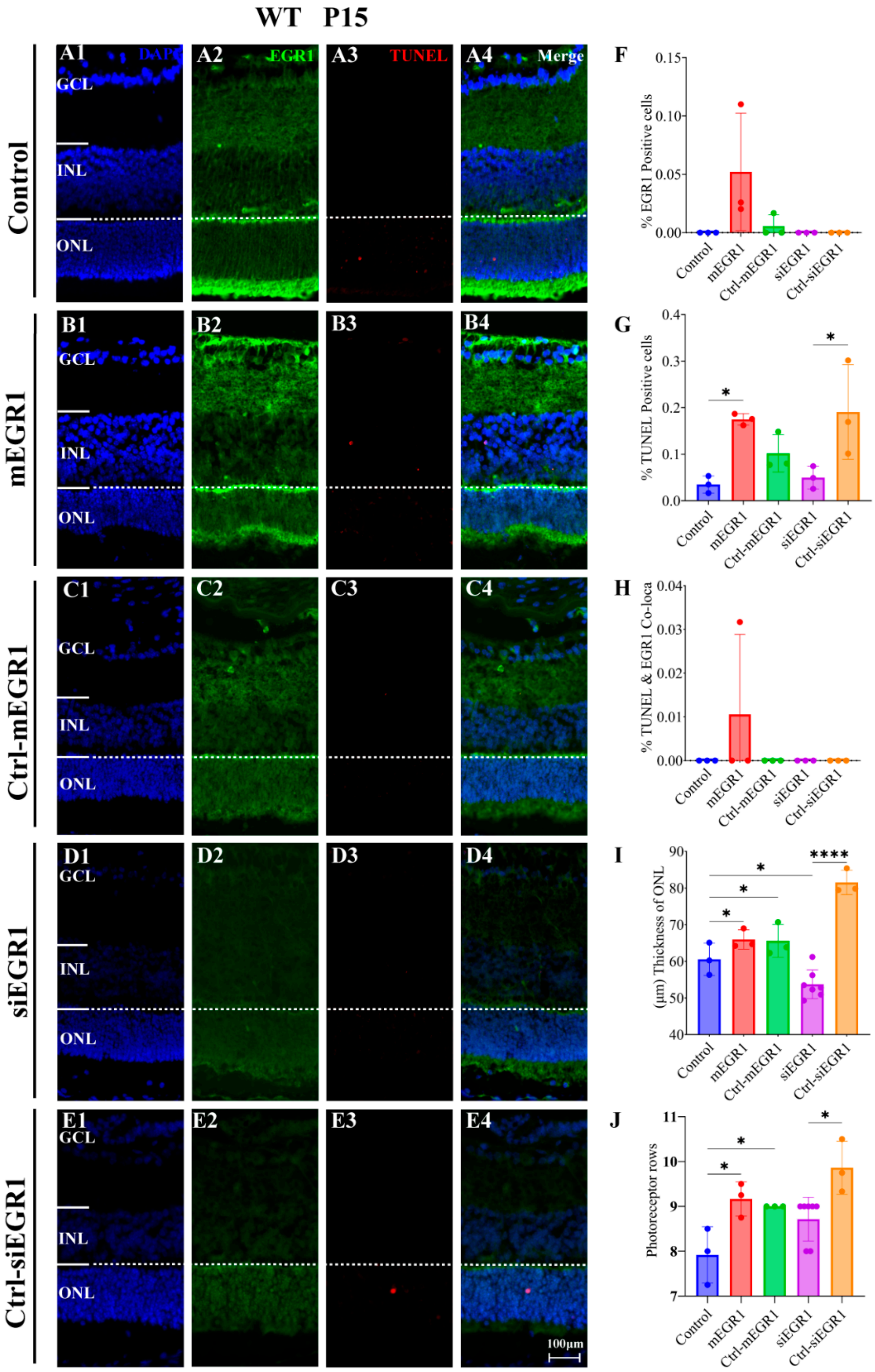

Figure S4. EGR1 expression is low in wild-type retina. At P5, wild-type (WT) mice were given a single suprachoroidal injection of an AAV carrying constructs targeting EGR1.

Expression of EGR1 (green) and TUNEL (red) in the retina at P15 WT mice was examined using immunofluorescence staining and TUNEL assay. Nuclei were counterstained with DAPI (blue*)*. (A-E) In WT retina overall there were only few EGR1 or TUNEL positive cells. (A1-A4) Untreated, control *rd1* retina. (B1-B4) Retina after treatment with AAV9-EGR1-GFP (7.68×10^{10} vg/ml). (C1-C4) Retina after treatment with a control construct for EGR1. (D1-D4) *rd1* retina treated with siRNA construct AAV9-EGR1-RNAi (3.676×10^{9} vg/ml). (E1-E4) Retina after treatment with a control construct for siRNA EGR1. (**F**–**J**) Quantification of (**F**) percentage of EGR1-positive cells in WT outer nuclear layer (ONL), (**G**)percentage of TUNEL-positive, dying cells in WT outer nuclear layer (ONL), (H) percentage of ONL cells displaying colocalization of TUNEL and EGR1, (**I**) thickness of ONL in µm, and (J) photoreceptor row counts. The images shown are representative for observations on at least three different specimens for each genotype. Error bars represent SD. ONL = outer nuclear layer, INL = inner nuclear layer, GCL = ganglion cell layer. Scale bar = 100 µm. * = $p < 0.05$; **** = $p < 0.0001$.

Table S1. Dual Luminescence Assay

SEAP	Ctrl-mEGR1+PAPR1/WT	12576.12601	11608.75306	10837.946
	Ctrl-mEGR1+PAPR1/MUT	7584.433455	9238.767989	10093.421
	mEGR1+PAPR1/WT	12576.12601	11608.75306	11869.991
	mEGR1+PAPR1/MUT	11931.17986	15155.81806	15386.365
GLUC	Ctrl-mEGR1+PAPR1/WT	11522.90736	11023.16937	10284.429
	Ctrl-mEGR1+PAPR1/MUT	7452.590527	7593.819383	8035.6154
	mEGR1+PAPR1/WT	6020.855219	6024.477112	7985.3705
	mEGR1+PAPR1/MUT	10508.9542	12804.83897	12956.938
Gluc/SEAP	Ctrl-mEGR1+PAPR1/WT	0.916252537	0.94955671	0.9489279
	Ctrl-mEGR1+PAPR1/MUT	0.982616641	0.821951519	0.7961241
	mEGR1+PAPR1/WT	0.478752774	0.518959882	0.672736
	mEGR1+PAPR1/MUT	0.880797568	0.844879433	0.8421052

Ctrl-mEGR1 +PAPR1 /WT	mEGR1 +PAPR1 /WT	Ctrl-mEGR1 +PAPR1 /MUT	mEGR1 +PAPR1 /MUT
0.916252537	0.478752774	0.982616641	0.880797568
0.94955671	0.518959882	0.821951519	0.844879433
0.948927904	0.672736001	0.796124061	0.842105198
